# Investigation of persuasive system design predictors of competitive behavior in fitness application: A mixed-method approach

**DOI:** 10.1177/2055207619878601

**Published:** 2019-11-01

**Authors:** Kiemute Oyibo, Julita Vassileva

**Affiliations:** University of Saskatchewan, Saskatoon, Canada

**Keywords:** Persuasive strategies, gamification, social influence, social comparison, social learning, reward, competition, intrinsic motivation, path model, fitness app

## Abstract

Fitness applications aimed at behavior change are becoming increasingly popular due to the global prevalence of sedentary lifestyles and physical inactivity, causing countless non-communicable diseases. Competition is one of the most common persuasive strategies employed in such applications to motivate users to engage in physical activity in a social context. However, there is limited research on the persuasive system design predictors of users’ susceptibility to competition as a persuasive strategy for motivating behavior change in a social context. To bridge this gap, we designed storyboards illustrating four of the commonly employed persuasive strategies (reward, social learning, social comparison, and competition) in fitness applications and asked potential users to evaluate their perceived persuasiveness. The result of our path analysis showed that, overall, users’ susceptibilities to social comparison (β_T_ = 0.48, *p* < 0.001), reward (β_T_ = 0.42, *p* < 0.001), and social learning (β_T_ = 0.29, *p* < 0.01) predicted their susceptibility to competition, with our model accounting for 41% of its variance. Social comparison partially mediated the relationship between reward and competition, while social learning partially mediated the relationship between social comparison and competition. Comparatively, the relationship between reward and social learning was stronger for females than for males, whereas the relationship between reward and competition was stronger for males than for females. Overall, our findings underscore the compatibility of all four persuasive strategies in a one-size-fits-all fitness application. We discuss our findings, drawing insight from the comments provided by participants.

## Introduction

Competition is one of the most effective socially oriented strategies employed in motivating behavior change in most persuasive applications on the market. It is employed mostly in the social context of gamification—in a non-game context^1^—to *intrinsically* motivate users to engage in the target behavior. Specifically, intrinsic motivation is “the doing of an activity for its inherent satisfactions rather than for some separable consequence” (p. 56),^2^ for example, engaging in an activity for the sake of the pleasure it provides. In persuasive technology interventions, it is recommended that the persuasive system design (PSD) should be aimed at fostering intrinsic motivation and user participation.^3,4^ In the education domain, for example, competition has been identified as one of the seven key intrinsic motivators—challenge, curiosity, fantasy, control, competition, cooperation, and recognition)^5,6^—employed in persuasive video games for education to facilitate learning and assimilation.^2^ In general, though the purpose of persuasive applications is not to promote competitive behavior among users, competition has become a necessary feature that is used to foster users’ interest and motivate them to continue to use a persuasive application for a long period of time.^7^ However, according to Ryan and Deci, “despite the observable evidence that humans are liberally endowed with intrinsic motivational tendencies, this propensity appears to be expressed only under specifiable conditions” (p. 58),^2^ which may be extrinsic or social. Thus, in persuasive technology research, based on the Oinas-Kukkonen and Harjumaa's PSD Framework (commonly used by persuasive technology researchers for designing and evaluating persuasive systems)^8^, it becomes pertinent for researchers to uncover the possible PSD predictors of users’ susceptibility to competition as a social strategy for motivating behavior change. Due to the natural human drive to outperform one another, competition has become one of the most powerful persuasive techniques in motivating users to achieve and sustain behavior change in various domains.^9^ In the context of our study, we set out to answer the overarching question, “If user A is susceptible to the persuasive strategy X in the PSD Framework, can we predict, with some degree of certainty, their level of susceptibility to the persuasive strategy of competition?” Answering this question will help us to predict the susceptibility of user A to competition if their persuasion profile (susceptibility to other persuasive strategies than competition) is known.^10^ To be more specific, in the context of our study, “If a user’s persuasion profile with respect to reward, social learning, or social comparison is known, can we or a persuasive system use it as a basis for determining their level of susceptibility to competition as a candidate socially oriented strategy for motivating behavior change?” Moreover, knowing the interrelationships among all four PSD strategies will unveil the compatibility of all strategies as a set of social strategies that can be implemented together in a persuasive application aimed at motivating behavior change in a social context. In a domain-independent study (Oyibo and Vassileva^11^), we found that these four strategies could be implemented in concert in a single persuasive application aimed at motivating behavior change in a social context. However, there is non-existent research to confirm this finding in domain-dependent contexts such as the fitness domain, in which competition is often used as a social strategy to motivate behavior change. For example, in the health domain, “will a use susceptible to reward strategy be also susceptible to competition strategy?” If “yes,” this will provide an empirical basis for the persuasive health application designer to implement reward elements in the application alongside competition as a composite persuasive technique for amplifying the performance of the target behavior. If “no,” for example, if the more susceptible a person is to reward strategy, the less likely they will be susceptible to competition strategy, then there is no empirical basis for implementing these two strategies together (as a combined persuasive strategy) as they are incompatible. The same reasoning applies to social learning and social comparison with respect to their relationship with competition. While the relationship between social comparison and competition seems to be obvious (since, by default, people tend to compare themselves with others while they compete), this is not the case with respect to social learning and competition (see the path model in Oyibo et al.^12^). Uncovering the latter relationship would serve as an empirical basis for the implementation of both strategies together in the same fitness application aimed at amplifying physical activity behavior change through competition.

To answer the above research questions in the health domain, we employed storyboards illustrating the four persuasive strategies (reward, social comparison, social learning and competition) to investigate the PSD predictors of competitive behavior in persuasive technology. Storyboarding is a common technique used in human–computer interaction design to illustrate system interfaces and their contexts of use. They depict an envisioned scenario of how a given application feature functions.^13^ Implementing and evaluating the effectiveness of persuasive strategies using storyboards facilitate the elicitation of useful responses from potential users of persuasive technology interventions, which can be used to inform the actual application design.^14^ Moreover, “storyboards provide a common visual language that individuals from diverse backgrounds can read and understand” (p. 2)^14^ and have been successfully applied in prior studies.^14,15^

In our study, we aimed to answer four main research questions using storyboards. They included (1) in the domain of health and fitness apps, can users’ susceptibility to competition be predicted based on their susceptibility to reward, social comparison, and social learning? (2) If yes, to what extent, and what does this mean in the context of fitness app design? (3) How does gender moderate the interrelationships among the three PSD predictors and the target construct (competition)? (4) Can the quantitative findings in the path model be confirmed by qualitative evidence found in participants’ comments? Having outlined the research questions addressed in this paper, we would like to note at this juncture that this paper is not about investigating every possible predictor (e.g., personality characteristics^16^) of users’ susceptibility to competition. Rather, as captured in the title, this paper is concerned with investigating other PSD predictors of competition by focusing on commonly employed persuasive strategies (reward, social learning, and social comparison) in the PSD Framework.^8^

To answer our research questions, we conducted Partial Least Square Path Modeling (PLSPM).^36^ The result of our path analysis showed that, overall, users’ susceptibilities to social comparison (β_T_ = 0.48, *p* < 0.001), reward (β_T_ = 0.42, *p* < 0.001), and social learning (β_T_ = 0.29, *p* < 0.01) predicted their susceptibility to competition, with our model accounting for 41% of its variance. Social comparison partially mediated the relationship between reward and competition, while social learning partially mediated the relationship between social comparison and competition. Further multigroup analysis showed that the models for males and females significantly different (*p* < 0.05) with respect to two mediated relationships. Users’ susceptibility to reward directly predicted social learning for females (β = 0.33, *p* < 0.001) but did not for males (β = 0.05, *p* = ns). However, users’ susceptibility to reward directly predicted competition for males (β = 0.34, *p* < 0.05) but did not for females (β = −0.04, *p* = ns). Specifically, social comparison had a stronger mediating effect on the relationship between reward and competition in the female model (variance accounted for (VAF)^8^ = 0.56) than in the male model (VAF = 0.21). VAF is the ratio of the indirect effect to the total effect. Moreover, the male model accounted for more variance of competition (53%) than the female model (39%). Overall, our findings showed that reward, social comparison, and social learning are good PSD predictors of competition. In other words, users’ susceptibility to competition can be predicted based on their susceptibility to all three persuasive strategies. In the context of fitness app design, our findings suggest that all four persuasive strategies can be employed side by side in a fitness app to motivate behavior change among our target audience. We discuss these findings and how they are supported by the qualitative evidence in participants’ comments.

The rest of the paper is organized as follows. Since there are no session numbers in the paper, replace with The next section focuses on related work, then the research method is presented, followed by the results of our path analysis and discussion of the findings.

**Table 1.** Persuasive strategies and their definitions.

Strategy	Definition
Reward	Reward is a persuasive strategy that supports the giving of incentives to users for the accomplishment of their goals in a persuasive application.
Social learning	Social learning is a persuasive strategy that allows users to observe the behaviors and achievements of other users in a persuasive application.
Social comparison	Social comparison is a persuasive strategy that allows users to view and compare their behaviors and achievements with those of other users of a persuasive application.
Competition	Competition is a persuasive strategy that leverages the human natural drive to outperform one another to motivate user engagement in a target behavior in a persuasive application.

Source: Oyibo K, Adaji I and Vassileva J.^18^

## Related work

A substantial number of studies have been carried out on social influence in the context of persuasive technologies. We review a cross-section of relevant papers, especially in the health domain. We have categorized the reviewed studies into two main sections: survey-based studies (quantitative and qualitative) and evaluations of persuasive health interventions in the physical activity domain. Table 1 shows the definition of the four persuasive strategies (reward, social comparison, social learning and competition), discussed in the paper.

### Survey-based studies

A number of quantitative studies on the effectiveness of social influence strategies have been conducted at the level of perception. Busch et al.^18^ developed a social influence scale called Persuadability Inventory to empirically measure users’ susceptibility to a number of socially oriented strategies such as social learning, social comparison, and competition. However, they did not investigate the interrelationships that exist among the constructs in the inventory.^11^ Subsequently, in our previous work (Oyibo et al.,^19,20^ we investigated the susceptibility of users to all four persuasive strategies and the moderating effect of gender, age, and culture. We found that, irrespective of the three demographic variables, users were susceptible to all four persuasive strategies. Similarly, in our work (Oyibo et al.,^21,22^ we investigated users’ susceptibility to Cialdini’s universal principles of social influence and how culture and gender moderate users susceptibility. Specifically, we found that, irrespective of culture^21^ or gender,^22^ participants were susceptible to social proof (also known as consensus). However, our studies did not focus on the interrelationships among the persuasive strategies investigated.

Based on the Persuadability Inventory,^18^ in our work (Oyibo et al.^11,12^), we proposed a model of competitive behavior in the general context of persuasive technology. The model comprises three hypothetical PSD predictors of users’ susceptibility to competition as a persuasive strategy—reward, social comparison, and social learning—which are commonly featured in persuasive applications on the market.^15,23^ We found that users’ susceptibility to reward and social comparison—but not to social learning—predicted their susceptibility to competition. However, our prior path modeling of competition was carried out in a domain-independent context. Hence, competition model has not been tested in a domain-specific context (e.g., health) to confirm its validity and replicability. Secondly, the model was not supported by qualitative evidence to strengthen its validity. Similarly, Stibe^24^ proposed a framework for identifying and leveraging the socially oriented design principles in the sharing of feedback among users. However, the study was focused on predicting users’ perceptions of the effectiveness of sociotechnical systems and users’ level of engagement in the sharing of Twitter's feedback tweets, and not predicting users’ susceptibility to competition. Stibe^25^ also proposed a similar framework for user engagement, which the author called the Socially Influencing Systems Framework. However, the framework was not specifically targeted at predicting users’ susceptibility to competition as a persuasive strategy, nor was Stibe’s study^24^ based in the health domain.

With regard to qualitative studies, in Orji et al.,^15^ we investigated the strengths and weaknesses of three socially oriented strategies (social comparison, competition, and cooperation) that could enhance or hinder the effectiveness of behavior change. Among other things, we found that social comparison/competition engages and challenges users to perform better in gamified applications, while providing fun and excitement that make the target behavior appear easier to do. On the other hand, we found that both strategies may cause anxiety by creating unnecessary tension and pressure, which could lead to loss of self-esteem and self-confidence.

### Persuasive health interventions

With respect to health interventions, a number of studies have investigated the effectiveness of social influence in promoting physical activity in experimental settings. Fujiki et al.^26^ evaluated a virtual racing game, *NEAT-o-Games*, which employed competition and reward strategies to encourage physical activity behavior. In their pilot experiment that took place over a period of few days, they found that players were engaged in the game by becoming more physically active while having fun at the same time. Specifically, their game “appeared to increase both the time and intensity of the players’ engagement in mild aerobic activities” (p. 19). Lin et al.^27^ developed a physical activity app, *Fish ‘n’ Steps*, and conducted a study to investigate the effect of a growing fish in a tank (incentive) and social influence (cooperation and competition) on users’ step counts over a 14-week period. They found that the gamified app, which provided users with feedback on their calories burned, personal progress, and ranking, resulted in a change in participants’ attitudes toward physical activity as well as an increase in their daily step count. Chen and Pu^28^ investigated the effectiveness of three gamification strategies (competition, cooperation, and a hybrid of both) in a mobile app called *HealthyTogether*. The app was connected to a Fitbit tracker that logged the number of daily steps and stairs taken. The authors found that all three strategies were successful, with cooperation being most effective, followed by the hybrid and then competition. Finally, Toscos et al.^29^ designed and evaluated a preventive mobile health app, called *Chick Clique*, which encouraged teenage girls through social influence (social comparison and competition) to exercise more. The authors found that *Chick Clique* changed “the isolated process of self-monitoring into a cooperative, supportive process where friends can share personal fitness information and give one another encouraging feedback.”^29^

### Summary and gaps in related work

Our review shows that most of the empirical studies on social influence (e.g., Oyibo et al.^21,22^) are based on investigating users’ susceptibility to persuasive strategies at the level of perception, using quantitative measures. However, there are limited mixed-method (quantitative and qualitative) studies on the interrelationships among socially oriented persuasive strategies, which are commonly employed in persuasive technologies aimed at behavior change.^15,23^ Moreover, Oyibo and Vassileva’s^11^ model of competitive behavior has not been investigated in a domain-specific context to confirm its validity or verify its replicability. Our study is aimed at bridging these gaps in the extant literature, using the fitness domain as a case study, storyboards to measure users’ susceptibility to persuasive strategies, and path analysis to model their interrelationships.

## Method

In this section, we present our research questions, hypotheses, the measurement instruments for the four persuasive strategies, and the demographic information of the participants who took part in the study.

### Research objective

**Figure 1.**
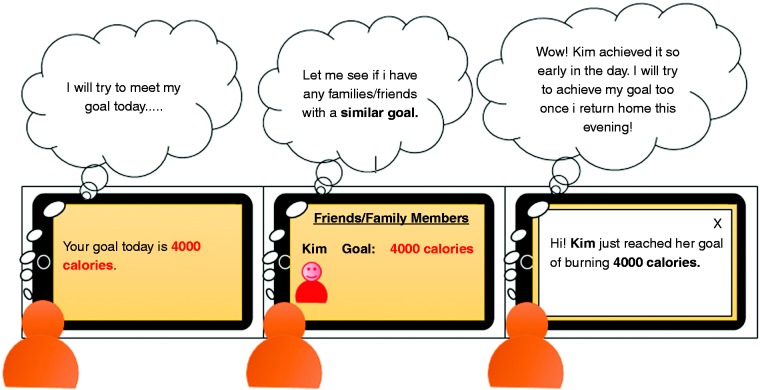
Storyboard illustrating social learning strategy in a fitness app.^18^

The aim of our study was to investigate the PSD predictors of users’ susceptibility to competition in fitness applications aimed at motivating behavior change, using storyboards illustrating four commonly employed persuasive strategies (reward, social learning, social comparison, and competition) in the persuasive technology domain. All four strategies were adopted from Oinas-Kukkonen and Harjumaa’s^30^ PSD model. We chose persuasive strategies these because they are often employed in persuasive applications for motivating behavior change in the health domain^31^ and they were part of the Persuadability Inventory proposed by Busch et al.^18^ Figure 1 shows an example of a storyboard illustrating the social learning strategy. Storyboards such as this have been used in prior studies,^32^ for example, in the eating domain^14,33^ to elicit qualitative feedback from participants on healthy eating. Specifically, our study aims to answer the following research questions (RQ's) with regard to fitness apps aimed at motivating behavior change:

- RQ1. Can users’ susceptibility to competition be predicted based on their susceptibility to reward, social comparison, and social learning?
- RQ2. If yes, to what extent, and what does this mean in the context of fitness app design?
- RQ3. How does gender moderate the interrelationships among the three PSD predictors and the target construct (competition)?
- RQ4. Can the quantitative findings in the path model be supported by qualitative evidence in participants’ comments?

### Research hypotheses

**Figure 2.**
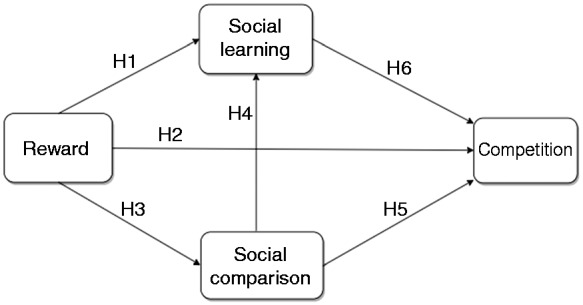
Hypothesized path model of competitive behavior in a fitness app.^11^

To answer the above research questions in the fitness domain, we adopted the model of competitive behavior proposed by Oyibo and Vassileva,^11^ shown in Figure 2, which is based on the following formally stated hypotheses:

**H_1_:** The higher the susceptibility of users to reward, the higher their susceptibility to social learning will be.

**H_2_:** The higher the susceptibility of users to reward, the higher their susceptibility to competition will be.

**H_3_:** The higher the susceptibility of users to reward, the higher their susceptibility to social comparison will be.

**H_4_:** The higher the susceptibility of users to social comparison, the higher their susceptibility to social learning will be.

**H_5_:** The higher the susceptibility of users to social comparison, the higher their susceptibility to competition will be.

**H_6_:** The higher the susceptibility of users to social learning, the higher their susceptibility to competition will be.

The first five hypotheses (H_1_–H_5_) were based on Oyibo and Vassileva’s^11^ model of competitive behavior in a domain-independent context. In the model, H_1_–H_5_ were validated. Moreover, the sixth hypothesis (H6) was based on the “finding that people who learn tested behaviors from others are more likely to perform them better than those who do not”.^8^ Thus, we hypothesize that the higher the susceptibility of users to social learning, the higher will be their susceptibility to competition as a persuasive strategy for motivating behavior change in a social context.

### Measurement instruments

Prior to administering the storyboards to participants, we presented them with a description of a fitness app prototype (which we call *Homex App*) to contextualize the study. The application description reads as follows:

Imagine you want to improve your personal health and fitness level. Given the challenges (e.g., time, cost, weather, etc.) associated with going to the gym regularly, the “Homex App” has been created, say by health promoters in your neighborhood, to support your physical activity.

Thereafter, with respect to each storyboard illustrating each persuasive strategy, the following set of questions was asked:

Imagine that you are using the Homex App presented in the storyboard above [see Figure 1] to track your physical activity, to what extent do you agree with the following statements:

1. This feature of the app would influence me.

2. This feature of the app would be convincing.

3. This feature of the app would be personally relevant to me.

4. This feature of the app would make me reconsider my physical activity.

Prior to responding to the above questions, participants were asked to study and identify the correct persuasive strategy in the storyboard from six options (the six investigated persuasive strategies) to increase the reliability of their responses and our findings. Responses associated with incorrectly identified persuasive strategies were treated as missing data points and replaced by the respective average scores during data analysis. We used the adapted version of the Perceived Persuasiveness Scale by Drozd et al.,^34^ which has been used by prior studies^35^ to measure the perceived persuasiveness of each strategy. The rating scale ranged from “Strongly Disagree (1)” to “Strongly Agree (7).”

### Participants

Our study was submitted to and approved by our university’s Research Ethics Board. Thereafter, it was posted on Amazon Mechanical Turk to recruit participants resident in Canada and the United States, who were compensated with US $1.50 each. Table 2 shows the demographics of participants after cleaning: 132 males, 95 females and 1 unidentified.

**Table 2.** Participants’ demographic information.

Criterion	(Female, Males, Others) = (95, 132, 1)
Age	18–24 (15, 23); 25–34 (40, 81, 1); 35–34 (24, 21); 45–54 (13, 3); 54+ (3, 4)
Education	Technical/trade school (16, 25); high school (16, 22, 1); bachelor’s (49, 58); master’s (12, 21); doctorate (1, 5); Others (1, 1)
Country of origin	Canada (38, 50, 1); United States (42, 56); Others (15, 26)
Continent of origin	North America (77, 96, 1); South America (3, 7), Europe (6, 7); Africa (0, 11); Asia (6, 7), Middle East (2, 3); Others (1, 1)

## Results

This section focuses on our path analysis, multigroup, total effect, effect size, and mediation analyses.

### Measurement model

Our path models were built using R’s (PLSPM^41^) package,^36^ starting with the assessment of the measurement models, in which we evaluated four criteria: indicator reliability, internal consistency reliability, convergent validity, and the discriminant validity of each construct. For each construct, the indicator reliability had an outer loading greater than 0.7, and the internal consistency reliability (DG.rho) was greater than 0.7. Similarly, the convergent validity for each construct (average variance extracted) was greater than 0.5. Finally, with respect to discriminant validity (based on the crossloading criterion), no particular indicator loaded higher on any other construct in the measurement model than the very construct it was meant to measure.^37^

### Data-driven path model

To verify our hypotheses, we built three models: the global, the male, and the female model. The gender-based models were as a result of our multigroup analysis, which showed that there was a significant difference between the male and female subgroups in our global population sample with respect to two of the hypotheses (*p* < 0.05).

#### Global model

**Figure 3.**
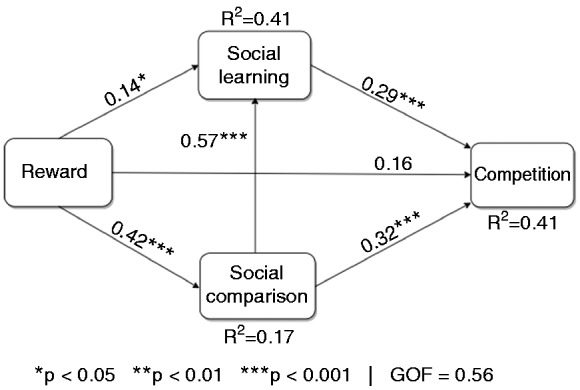
Global model of users’ susceptibility to competition in a fitness app.

Figure 3 shows the global model for the general population sample. The model is characterized by three parameters: (1) the coefficient of determination (*R*^2^), which represents the amount of variance of each endogenous construct explained by its corresponding exogenous constructs^38^; (2) The goodness of fit (GOF), which measures the predictive performance of the model, that is, how well the model fits its data^36^; and (3) the path coefficient (β), which indicates the strength of the relationship between one construct and another. In the global model, the three PSD predictors accounted for 41% of the variance of competition, with the model’s GOF being 56%. Moreover, five of the six relationships (H_2_, H_3_, H_4_, H_5_, and H_6_) were statistically significant. First, reward positively predicted social learning (β = 0.14, *p* < 0.05) and social comparison (β = 0.42, *p* < 0.001). Second, social comparison positively predicted social learning (β = 0.57, *p* < 0.001) and competition (β = 0.32, *p* < 0.001). Third, social learning positively predicted competition (β = 0.29, *p* < 0.001). However, the direct relationship between reward and competition (β = 0.16, *p* = ns) was non-significant.

#### Gender-based models

**Figure 4.**
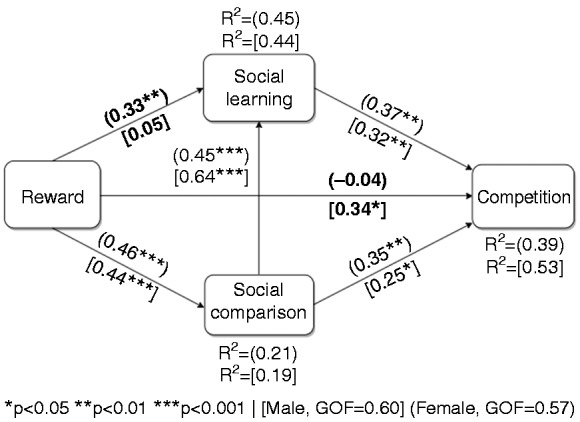
Gender-based models of users’ susceptibility to competition (the bold path coefficients indicate there is a significant difference (*p* < 0.05) between the male group (*n* = 132) and the female group (*n* = 95)).

Figure 4 shows the male and female models combined in one path model for brevity and ease of comparison. The parameters characterizing the male and female models are enclosed in square and circular brackets, respectively. For the most part, the submodels mirrored the global model, except that the male model (*R*^2^ = 51%) accounted for more of the variance of competition than the female model (*R*^2^ = 39%). Moreover, the multigroup analysis showed that there was a significant difference (*p* < 0.04) between males and females with respect to the relationship between (1) reward and social learning, which was stronger for the female (β = 0.33, *p* < 0.01) than the male group (β = 0.05, *p* = ns); and (2) reward and competition, which was stronger for the male (β = 0.34, *p* < 0.01) than the female group (β = −0.04, *p* = ns).

#### Effect Size, total effect, and mediation

Table 3 shows the effect sizes ( *f* ^2^) of the exogenous constructs on the endogenous constructs, the total effect of the three PSD predictors on the target construct (competition), and the mediating role played by social learning and social competition in the model. Effect size is a measure of the substantive impact of one construct on another.^38^ Social learning had a weak effect size on competition ( *f* ^2^ = 0.11), which cut across both genders and was stronger for the female ( *f* ^2^ = 0.12) than the male group ( *f* ^2^ = 0.17). Social comparison had a small effect on competition ( *f* ^2^ = 0.06) but a medium effect size on social learning ( *f* ^2^ = 0.18). Both effect sizes were stronger for the male group ( *f* ^2^ = 0.04 and 0.23, respectively) than for the female group ( *f* ^2^ = 0.01 and 0.18, respectively). Overall, reward had an effect size, ranging from none to medium, on social learning and competition. The largest effect size of reward ( *f* ^2^ = 0.11 for the male group) and the smallest, which was non-existent ( *f* ^2^ = −0.04 for the female group) was on competition. With respect to total effect, all three PSD predictors had a significant effect on competition. However, social comparison (β_T_ = 0.48, *p* < 0.001) and reward (β_T_ = 0.42, *p* < 0.001) had a stronger total effect (about 0.45 overall and at the subgroup level) than social learning (about 0.33) on competition. Finally, with respect to mediation, social comparison, overall and regardless of gender, partially mediated the effect of reward on social learning and competition. Specifically, mediation by social comparison (of the effect of reward on competition) was strongest for the female (VAF = 0.56) and weakest for the male group (VAF = 0.21).

**Table 3.** Effect Size and mediation based on the global model. VAF = variance accounted for; 0.02 ≤ VAF < 0.80 represents partial mediation; VAF ≥ 0.80 represents full mediation; 0.02 ≤ ES < 0.15 represents a small effect size; 0.15 ≤ ES < 0.35 represents a medium effect size; ES ≥ 0.35 represents a large effect size (Hair et al.^39^). ‘–’ defines no mediation.

	Global	Male	Female
**Effect size ( *f* ^2^)**			
Social learning → Competition	0.11	0.07	0.12
Social comparison → Competition	0.06	0.04	0.01
Social comparison → Social learning	**0.18**	**0.23**	**0.18**
Reward → Social learning	0.02	0.01	0.09
Reward → Competition	−0.02	0.11	−0.04
**Total effect (β_T_, *p* < 0.001)**			
Reward → Competition	0.42	0.44	0.46
Social comparison → Competition	0.48	0.45	0.45
Social learning → Competition	0.29	0.32**	0.37*
**Mediation (VAF)**			
Reward → Social learning → Competition	0.16	–	–
Reward → Social comparison → Competition	0.36	0.21	0.56
Reward → Social comparison → Social learning	0.38	0.45	0.30
Social comparison → Social learning → Competition	0.29	0.32	0.37

*Note:* Bold values represent strong effect sizes.

### Qualitative comments supporting the validated hypotheses

We analyzed participants’ comments to investigate whether the positive (quantitative) interrelationships between the path model’s PSD predictors (reward, social learning, and social comparison) and competition could also be found in their comments. Tables 4 and 5 show the result of our analysis, which comprise participants’ qualitative comments (on all four persuasive strategies) and their quantitative persuasion profiles (average scores in all four strategies). Table 4 shows positive (motivating) comments on all four strategies, and illustrates the participants’ persuasion profiles, while Table 5 shows a cross-section of negative (demotivating) comments on all four strategies, and participants’ persuasion profiles. In the following section, we discuss the comments in each of the tables in the light of the interrelationships among the four constructs in the path model.

**Table 4.** Positive participant comments supporting the relationships between the model’s constructs (motivators of competitive behavior). RW = Reward, SC = Social Comparison, SL = Social Learning, CT = Competition. The profile represents a participant’s average scores in the rating of the storyboards illustrating the persuasive strategies in terms of perceived persuasiveness. Overall persuasion profile average scores (RW, SC, SL, CT) = (4.13, 3.50, 3.71, 3.93).

Reward	Social Comparison	Social Learning	Competition	Profile
I would want to reach my goals and get rewarded.	I would want to do better like my friends.	If friends and family are working hard it will make me want to as well.	I would want to beat my friends and family.	P18:RW = 5.75SC = 4.75SL = 5.50CT = 5.75
Everyone needs some form of incentive or motivation even if it is self-motivation to reach their goals and reward is very compelling.	Similar to competitive leader boards being able to compare yourself to friends for a nice push is great.	I am fairly competitive and being able to see what my friends are doing may help push me even further to accomplish my goals.	I am competitive and seeing a leaderboard would push me to be on top week in and week out setting my limits to higher bounds.	P127:RW = 4.50SC = 4.75SL = 5.25CT = 5.75
I would enjoy getting rewards when meeting my goals and the app would be relevant in this way.	Comparing how I'm doing with my friends would help me to motivate myself when down.	I would love comparing myself to friends and motivating each other!	Competition would spur me on too to do better!	P140:RW = 5.00SC = 6.00SL = 5.00CT = 5.00
Again, the idea surrounding goal-setting and the rewards would vary by individual and for myself this does not get me excited to do a home workout but may have a slight influence in getting me to be more active.	This feature would hold me accountable to myself not to fall behind and therefore I would indeed look to make the necessary changes to my routine in order not to fall behind.	I like how this introduces an element of social competition as a means of motivation to continue and follow through with a workout plan as I am a competitive person this would work to motivate and influence me to not slack off or take unnecessary time away from physical activity.	This would motivate and influence me to push harder every day to achieve the top rank (or attempt to) therefore this level of competition does indeed convince influence.	P172:RW = 4.25SC = 6.00SL = 5.00CT = 6.00

**Table 5.** Participant comments supporting the interelationships among the model’s constructs (demotivators of competition). RW = Reward, SC = Social Comparison, SL = Social Learning, CT = Competition. The profile represents a participant's average scores in the rating of the storyboards illustrating the persuasive strategies in terms of perceived persuasiveness. Overall persuasion profile average scores (RW, SC, SL, CT) = (4.13, 3.50, 3.71, 3.93).

Reward	Social Comparison	Social Learning	Competition	Profile
I don't think that bonus points would really affect me because I work out for the enjoyment and strength that I gain.	I don't think that I would be motivated by comparing myself to what others have done.	While it would be nice to see what other people are doing, I don't think that would make me feel more motivated because we all have different goals.	I'm not very competitive.	P60:RW = 4.00SC = 3.00SL = 2.00CT = 1.00
Don't care to get useless points, unless there is some real reward to be gained.	I don't like competition/comparison.	I hate comparison with other people. It's a silly.	I don't like competition/comparison.	P27:RW = 3.50SC = 1.00SL = 3.55CT = 1.00
Getting points as a reward would not motivate me.	I do not want to be compared to other people.	This wouldn't help me.	I would not want other people seeing how I am doing.	P2:RW = 3.00SC = 3.00SL = 3.00CT = 2.00
Reward features don't really interest me at all. I'm more focused on data and physical results of my workout than gaining points or a score. It just isn't my kind of thing, sorry.	Again, I'm not a social person so I wouldn't have any friends to compare my results with. However, I could see this being useful to others, but it is just a little too "social media-ish" for me, sorry.	This feature wouldn't be useful to me, but I'm a little different when it comes to social stuff. I feel that such a feature would be incredibly useful for most people, though. I don't engage in social media or talk with people very often…	I'm not the competitive type. I simply workout for my own results, not to obtain virtual rewards. This feature doesn't interest me.	P118:RW = 1.50SC = 1.75SL = 1.50CT = 3.50

## Discussion

We have proposed a model for predicting the susceptibility of users to competition as a persuasive strategy for motivating exercise behavior change in fitness apps. The global model accounted for about 40% of the variance of users’ susceptibility to competition. Table 6 shows a summary of the supported and unsupported hypotheses at the global and subgroup levels. All six hypotheses were supported at the global level, while only five were supported at the subgroup levels. We discuss the validation of all the hypotheses, the effect sizes, total effects, and mediations in the light of our research questions.

**Table 6.** Supported and unsupported hypotheses (relationships) in the global and gender-specific models.

Hypothesis	Relationship	Global	Male	Female
H_1_	Reward → Social learning	✓	×	✓
H_2_	Reward → Competition	✓	✓	×
H_3_	Reward → Social comparison	✓	✓	✓
H_4_	Social comparison → Social learning	✓	✓	✓
H_5_	Social comparison → Competition	✓	✓	✓
H_6_	Social learning → Competition	✓	✓	✓

“✓” indicates supported hypothesis (significant relationship); “×” indicates unsupported hypothesis (non-significant relationship).

### Validation of hypotheses

This section focuses on the validation and non-validation of the six hypotheses, taking the predictive persuasive strategies one at a time.

#### Validation of reward-related hypotheses (H_1_, H_2,_ and H_3_)

All the reward-related hypotheses (H_1_–H_3_) were supported, except for H_1_ in the male model and H_3_ in the female model. First, our path analysis showed that H_1_ was supported in the global and female model. However, it was not supported in the male model. A plausible explanation for this is that the relationship between reward and social learning was more partially mediated by social comparison in the male model (VAF = 0.45) than in the global model (VAF = 0.38) and female model (VAF = 0.30), as shown in Table 3. That said, given that H_1_ was validated in the global and female models, we can conclude that, overall, H_1_ was validated. This suggests that the higher the susceptibility of users, especially females, to reward as a persuasive strategy, the more likely they are to be susceptible to social learning as a socially oriented strategy for motivating exercise behavior change. Second, our path analysis showed that H_2_ was supported in the global and male models but not in the female model. Just as in the case of H_1_, a plausible explanation behind the non-validation of H_2_ in the female model is that the relationship between reward and competition was most partially mediated by social comparison in the female model (VAF = 0.56) compared with the global model (VAF 0.36) and male model (VAF = 0.21). Therefore, as in the case of H_1_, we can conclude that, overall, H_2_ was validated. This suggests that the higher the susceptibility of users, especially males, to reward as a persuasive strategy, the more likely they are to be susceptible to competition as a socially oriented strategy for motivating exercise behavior change. Finally, H_3_ was supported regardless of the model (global or subgroup) in question. This suggests that the higher the susceptibility of users to reward as a persuasive strategy, the more likely they are to be susceptible to social comparison as a socially oriented strategy for motivating exercise behavior change. Putting it all together, at a high level, we can conclude that a user that is susceptible to reward is very likely to be susceptible to social learning, social comparison, and competition, with social comparison partially mediating the relationship between reward and social learning and between reward and competition. Most of these findings in the physical activity domain are consistent with those found by Oyibo and Vassileva^11^ in the original model of competitive behavior in a non-domain-specific context.

#### Validation of social comparison-related hypotheses (H_4_ and H_5_)

All the hypotheses related to social comparison as an exogenous construct (H_4_ and H_5_) were supported regardless of the model. The validation of H_4_ and H_5_ suggests that the higher the susceptibility of users to social comparison, the more likely they are to be susceptible to social learning and competition, with social learning partially mediating the relationship between social comparison and competition, as shown in Table 3. This means that, in a gamified fitness application, for example, users who have a tendency to compare their performance and achievements with others’ are more likely to respond to social learning (observe and act on the knowledge of the performance and achievements of others) and be more competitive than users who do not have the tendency of comparison. Moreover, regardless of gender, the effect size of social comparison on social leaning (medium) was larger than on competition (small). This means, in a practical context, that users who are susceptible to social comparison are more likely to respond to social learning strategy than they will to competition strategy.

#### Validation of social learning-related hypotheses (H_6_)

As shown in Table 6, the hypothesis on the relationship between social learning and competition (H_6_) was supported regardless of the gender-specific model. The validation of H_6_ suggests that the higher the susceptibility of users to social learning, the more likely they are to be susceptible to competition as a socially oriented strategy for motivating exercise behavior change. In other words, a user who engages in observing and learning from other users in a gamified fitness application is more likely to be competitive in the game. This is a new finding in the model of competitive behavior. In the original model, Oyibo and Vassileva^11^ based on a domain-independent context, we found that the relationship between social leaning and competition was non-significant (see Table 7).

### Confirming the interrelationships among the model’s constructs (persuasive strategies) using participants’ comments

In this section, we provide qualitative evidence to support the quantitative findings. Table 4 and Table 5 show participants’ persuasion profiles and comments on the perceived pervasiveness of the four strategies illustrated on storyboards. Basically, the comments confirm the positive interrelationships among the four constructs in the path model. For example, as shown in Table 4, P18 and P127 scored above the average values and the neutral value of 4 in all four persuasive strategies. This indicates that both participants are susceptible to all four strategies. Moreover, their susceptibility to all four strategies (as evident in their persuasion profiles) is confirmed by the respective comments their provided on the perceived persuasiveness of the four strategies. For instance, P127, with a reward score of 4.5, had a positive view on the reward strategy, as evident in his comment, “Everyone needs some form of incentive or motivation even if it is self-motivation to reach their goals and reward is very compelling.” His positive view on reward seems to have propagated to the other three strategies on which he also had positive views. For example, with respect to social learning and social comparison, his positive views, indicating susceptibility, include “I am fairly competitive and being able to see what my friends are doing may help push me even further to accomplish my goals” and “Similar to competitive leader boards being able to compare yourself to friends for a nice push is great,” respectively. These positive views on all three PSD predictors can be viewed as a motivator of competitive behavior for P127, as evident in his comment on competition, “I am competitive and seeing a leaderboard would push me to be on top week in and week out setting my limits to higher bounds.”

On the other hand, we see that participants’ negative comments on the perceived persuasiveness of the strategies cut across all four strategies (also evident in their susceptibility profiles). In other words, if a user is not susceptible to any of the PSD predictors of competition, it is unlikely that they will be competitive in a social context. For example, in Table 5, P60, who scored below the respective averages in all four strategies, tended to be less or not motivated by reward, as evident in her comment, “I don't think that bonus points would really affect me because I work out for the enjoyment and strength that I gain.” Similarly, P60 tended to be less or not motivated by social learning and social comparison, as evident in the comments, “While it would be nice to see what other people are doing, I don't think that would make me feel more motivated because we all have different goals” and “I don't think that I would be motivated by comparing myself to what others have done,” respectively. The negative comments on the three PSD predictors—reflecting P60’s non-susceptibility to any of the three predicting persuasive strategies—suggest she is not a competitive person. This is evident in the comment “I'm not very competitive.”

In sum, we can conclude—in respect of answering our fourth question—that the quantitative findings in the path model were supported by the qualitative evidence in the participants’ comments.

### Comparison of the current study’s with Oyibo and Vassileva’s^11^ findings

We compared the relationships in our model/submodels (current: C) with those of Oyibo and Vassileva’s (previous : P) to see how the findings in both studies are similar and differ. The result of the comparison is shown in Table 7. Overall, that is, at the global level, four of the relationships are similar in both models; however, two of the relationships (reward → social learning and social learning → competition) are different. Specifically, the latter two relationships are significant in our current domain-specific model (C) but are non-significant in our previous domain-independent model (P). With respect to the male submodels, all the relationships are similar (significant) in both C and P, except for reward → social learning, which is only significant in C. Finally, with respect to the female submodels, two of the relationships are similar (reward → social comparison and social comparison → social learning), while the other four differ. Overall, at the global and male-group levels, 66.6% of the relationships are replicated, while at the female-group level, only 33.3% of the relationships are replicated. This shows that the model of competitive behavior in a domain-specific context (such as the fitness domain) differs from that in the domain-independent context with respect to some of the relationships and gender. Thus, there is a need to validate the model in domain-specific contexts. to uncover the moderating effect of domains on the interrelationships in the competition model.

**Table 7.** Comparison of the supported and unsupported hypotheses (relationships) of the current study with those of a previous study.

		Global		Male		Female	
Hypothesis	Relationship	C	P	C	P	C	P
H_1_	Reward → Social learning	✓	×	×	✓	✓	×
H_2_	Reward → Competition	✓	✓	✓	✓	×	✓
H_3_	Reward → Social comparison	✓	✓	✓	✓	✓	✓
H_4_	Social comparison → Social learning	✓	✓	✓	✓	✓	✓
H_5_	Social comparison → Competition	✓	✓	✓	✓	✓	×
H_6_	Social learning → Competition	✓	×	✓	×	✓	×

Note: C = current study in a domain-specific context (fitness domain); P = previous study by Oyibo and Vassileva^11^ in a domain-independent context.

“✓” indicates supported hypothesis (significant relationship); “×” indicates unsupported hypothesis (non-significant relationship).

### Summary and implication of findings

Based on the results we have presented and the findings discussed, we have provided answers to our research questions. With respect to our first research question, based on our path analysis, we can conclude that, in the domain of fitness apps, users’ susceptibility to competition as a persuasive strategy for motivating behavior change can be predicted based on their susceptibility to reward, social comparison, and social learning. With respect to our second research question, this implies that users who score high on any of the three predictive persuasive strategies in their persuasion profile, are likely to be susceptible to competition as a persuasive strategy for motivating their behavior change. On the flip side, users, who score low on any of the three predictive persuasive strategies in their persuasion profile, are unlikely to be susceptible to competition as a persuasive strategy. Thus, all four persuasive strategies, based on their significant interrelationships, can be implemented together in a fitness application to motivate users susceptible to any of the four persuasive strategies. A typical example would be a gamified application,^11^ equipped with a *reward*-based leaderboard, which enables users to visualize their and *learn about* others’ exercise performance (e.g., calories burned) and achievements, and *compare* and *compete* with one another. For example, in such an app, users at the top of the leaderboard can be offered incentives such as points, badges, levels, medals, etc., to motivate them to become even more competitive in the performance of the target behavior. However, to foster privacy, the leaderboard could be implemented in an anonymous fashion to prevent one user from uncovering the true identity of another who may not want to be identified, especially when they are underperforming. Regarding the third research question, based on our multigroup analysis, we conclude that gender moderates the interrelationships between the three PSD predictors and competition. Specifically, the relationship between reward and social learning is stronger for females than for males, while the relationship between reward and competition is stronger for males than for females. Finally, regarding the fourth research question, based on our qualitative analysis, we conclude that the quantitative findings based on the path model are supported by the qualitative evidence in participants’ comments, as shown in Table 4 and Table 5.

### Contributions

Our contribution to the body of knowledge is that, using a mixed-method approach in the context of a fitness application, we showed that users’ susceptibility to competition (an intrinsic motivator of exercise behavior) can be predicted based on their level of susceptibility to reward, social learning, and social comparison, all of which are commonly employed features of persuasive applications on the market. More specifically, our model replicated Oyibo and Vassileva’s^11^ original model of competitive behavior (in a domain-independent context) in the fitness domain, suggesting that the latter model holds true in domain-specific contexts such as health. Our study is the first to replicate the model in a domain-specific context, however, our study found some differences between the contexts. Specifically, in the fitness domain, we found that the relationship between users’ susceptibility to social learning and their susceptibility to competition was significant. (This is contrary to Oyibo and Vassileva’s^11^ finding in the domain-independent context in which the relationship in question was non-significant.) Secondly, we showed how gender moderates the relationship between reward and competition, and that between reward and social learning in the path model. Specifically, we showed that the relationship between reward and competition was stronger for males than for females, while the relationship between reward and social learning was stronger for females than for males. Thirdly, we showed that social comparison partially mediated the relationship between reward and competition, while social learning partially mediated the relationship between social comparison and competition. Finally, we demonstrated that all four persuasive strategies were compatible in a one-size-fits-all gamified fitness application aimed at motivating exercise behavior change.

### Limitations and future work

Our study has a number of limitations. First and foremost, it is based on self-report and storyboards in the context of a fitness application and not an actual application. This may have compromised the generalizability of our findings to the application domain, as well as to other health domains than fitness application. In other words, the significant relationships between some of the persuasive design strategies are at the level of perception rather than actual usage of a persuasive application, in which users’ susceptibility to each strategy is objectively measured. Thus, in the context of experimental studies that employ an actual persuasive application, some of the findings in this paper may differ. The second limitation of our study is that most of the participants were from Canada or the United States. This may limit the generalizability of our findings to other demographics, for example, other countries, cultures, continents, and so forth. The third limitation of our study is that we did not investigate the moderating effect of other key demographic such as age, education, Internet experience, and so on. The fourth limitation is that we did not investigate other possible PSD predictors of competition than reward, social learning, and social comparison. Finally, the fifth limitation of our work is that, while we refer to reward, social learning, and social comparison as predictive persuasive strategies in our PLSPM, we cannot, for certain, claim or confirm that users’ susceptibility to these strategies is the cause of their susceptibility to the competition strategy. We say this because the significant relationships between any two of the four persuasive strategies in the path model could be just correlational. As we have heard time and time again, “correlation is not causality,” and this applies to the findings we have presented in this paper. Moreover, the significant interrelationships we found among the four persuasive strategies could be due to participants’ underlying personality characteristics, which we did not investigate in this study. For example, people who think they are very independent of external and/or social influence might report that they are not susceptible to any of the four strategies, while those that think otherwise might report that they are susceptible to all of them. Thus, there may be an underlying personality characteristic (see Oyibo and Vassileva^16^) that may be responsible for the significant relationship between any two of the persuasive strategies, which we did not account for in our model.

In future work, we look forward to addressing some of these limitations, including investigating our model in a real-life application context, and among other demographics. We will also investigate the moderating effect of demographic variables other than gender, and additional possible PSD predictors of competition (e.g., goal-setting and self-monitoring, which are among the commonly researched and employed persuasive strategies in persuasive health applications^23,39,40^).

## Conclusion

In this paper, we presented a path model of users’ susceptibility to competition as a socially oriented persuasive strategy for motivating behavior change in the fitness domain. Our model has shown that users’ susceptibilities to reward, social learning, and social comparison are significant PSD predictors of their susceptibility to competition, with our model accounting for about 40% of the variance of competition. Moreover, our model has shown that there are significant gender differences in the competition model for males and females. Specifically, the relationship between reward and social learning is stronger for females than for males. However, the relationship between reward and competition is stronger for males than for females. Our findings, overall, suggest that, based on the significance of the interrelationships in the overall competition model, if users are susceptible to any of the three predictive persuasive strategies (reward, social learning, and social comparison), they are more likely to be susceptible to competition as well. Thus, in a one-size-fits-all fitness application, all four strategies could be implemented in concert and leveraged as persuasive strategies for motivating behavior change among potential users susceptible to any of the four strategies. In future work, we intend to validate the interrelationships in the competition model among participants from other demographics as well as in a real-life application context.
